# Does Cancer Type Influence the Impact of Recurrence? A Review of the Experience of Patients With Breast or Prostate Cancer Recurrence

**DOI:** 10.3389/fpsyg.2021.635660

**Published:** 2021-06-29

**Authors:** Ross James Stewart, Gerald Michael Humphris, Jayne Donaldson, Susanne Cruickshank

**Affiliations:** ^1^Faculty of Health Sciences and Sport, University of Stirling, Stirling, United Kingdom; ^2^School of Medicine, University of St Andrews, St Andrews, United Kingdom; ^3^Royal Marsden National Health Service Foundation Trust, London, United Kingdom

**Keywords:** breast cancer, oncology, prostate cancer, integrative review, quality of life, cancer recurrence

## Abstract

**Objective:** Patients will experience a plethora of issues when faced with a recurrence of their cancer. It is unclear if cancer type is a significant factor in how recurrence is experienced by an individual. The aim of the current review is to explore the evidence base and summarise the experiences of patients specifically with a recurrence of breast or prostate cancer (the most common for women and men, respectively) and then provide a comparison of these experiences. These experiences include the physical, psychological and psychosocial issues that arise at this time.

**Methods:** A systematic search was conducted of studies published between January 1994 and April 2019. Due to the mix of research designs used previously in the literature, this review was conducted in an integrative manner; allowing for inclusion of diverse research designs. Results were synthesised narratively, with data categorised according to physical, psychological, and psychosocial indices of quality of life. The review protocol was registered in the international database of prospective systematic reviews in health and social care- (CRD42019137381).

**Results:** Fifteen breast cancer and six prostate cancer articles were identified, each reporting one relevant study. Patients reported several negative issues at the time of a breast or prostate cancer recurrence. Similarities were found between cancer types, with physical problems such as fatigue, psychological issues including anxiety and depressive symptoms, and psychosocial concerns such as issues with healthcare professionals common in both cancers. Certain findings were inconsistent across studies, with some experiences differing between studies rather than due to cancer type.

**Conclusions:** Differences in the experience of recurrent cancer appear to be more heavily influenced by individual factors, rather than cancer type. Findings are confounded by gender; and should be considered preliminary. Effects of recurrence should be studied in samples where cancer type and gender are not confounded. Concerns are raised about available study quality and differing outcome measures in this interpretation. Care and support of the individual at the time of a cancer recurrence is a key focus. Future research suggestions with implications for clinical practise are included.

**Systematic Review Registration:** PROSPERO 2019 CRD42019137381.

## Introduction

Individuals will experience a range of negative consequences when faced with a cancer diagnosis, and the significance of an initial diagnosis is well-established in the literature (Schouten et al., 2019). However, it has been suggested that cancer recurrence may have a more significant impact on the individual than the initial disease as it often represents a more serious diagnosis (Step and Ray, 2011), particularly if the recurrence is not local. Consequently, the fear that cancer will recur is a common issue (Lebel et al., 2016); and has been addressed through psychological interventions (Chen et al., 2018).

In accordance with the negative consequences of a recurrence of cancer, some previous research has sought to capture the experience of patients at this time. A meta-ethnography (Wanat et al., 2016) reviewed qualitative studies involving recurrent cancer patients. This added to an earlier narrative review (Vivar et al., 2009) that summarised findings from varying study designs describing the impact on family members as well as the patient. Both reviews highlighted a complex range of issues patients face when dealing with a recurrence in relation to their physical well-being, emotional state, relationships- both personal and with healthcare professionals, as well as adjusting to new uncertainty and coming to terms with their own mortality.

In the UK, breast cancer is the most common malignancy in females, and prostate cancer the most common in males (Cancer Research, U. K., 2017b,c), and naturally the manner in which recurrence manifests will differ. In prostate cancer a patient may be diagnosed with biochemical recurrence. This refers to rising levels of prostate specific antigen (PSA) in the blood, but patients may not experience local or distant recurrence for some years after this (Artibani et al., 2018). In comparison, breast cancer recurrence may be identified in a manner similar to initial diagnosis, that is physical symptoms (Cancer Research, U. K., 2017a). With cancer in general it is known that several factors (including cancer characteristics) are important in understanding the well-being of patients (Schouten et al., 2019), but it is suggested that recurrence is a unique experience (Wanat et al., 2016) and yet there is little understanding of the effect of cancer type on how a recurrence affects the well-being of patients. Whilst being very common, these cancers manifest very differently, and as such may be a more useful point of comparison when establishing differences in reactions to recurrence than cancers with a more similar physical manifestation and treatment profile.

The aim of this review is to explore the existing literature in order to clarify if cancer type will influence the perceived impact of recurrence. By specifically examining prostate and breast cancer this review will explore highly prevalent, physically contrasting, and predominately gender based cancers; leading to a pertinent and multifaceted comparison. This will be conducted by summarising studies that evaluated the experiences of patients specifically with a recurrence of breast or prostate cancer; and then comparing these. For the purposes of this review, the patient experience refers to physical, psychological, and psychosocial issues that arise after a recurrence of cancer that may impact quality of life. For clarity, these experiences will relate to outcomes from studies assessing patient-reported levels of physical, psychological, and psychosocial indices of quality of life (QoL).

By addressing the question of cancer type potentially influencing the impact of recurrence it is suggested that findings from this review will help to develop a wider understanding of recurrence, highlighting differences (or the lack thereof) in personal reactions to a recurrence of these cancers. It is hoped that this will contribute knowledge to clinical care settings with implications for healthcare professionals treating patients with these cancer types. This includes professionals involved in regular personal care with these patients, such as cancer nurses. This is particularly important as, for some time, the NHS has outlined the need for a comprehensive approach to healthcare, in particular “person-centred” care- identifying the individual's wider well-being as crucial to their overall recovery, thereby providing a more personalised experience than in the past (Howe, 2020).

## Methods

In the literature, studies relevant to cancer recurrence feature a variety of research designs. Therefore, the current review was conducted in an integrative manner. This was considered a suitable method as it allows for inclusion, and deep understanding of diverse research designs (Hopia et al., 2016). The review was implemented in a systematic manner conforming to the methodological approach by Whittemore and Knafl (2005) that reduces the likelihood of biases and errors (Souza et al., 2010). The review protocol was registered in the international database of prospective systematic reviews in health and social care- PROSPERO 2019 CRD42019137381.

### Search Strategy

Following the rationale of previous reviewers (Wanat et al., 2016) who highlight that there have been significant changes in treatments for cancer and within healthcare services, it was decided to restrict the search from January 1994 to April 2019. Four electronic databases were searched: PsycInfo, CINAHL complete, Medline, and Pubmed. The following search terms were used:

- cancer^*^ or carcinoma^*^ or malignan^*^ or tumour or tumour or neoplasm^*^
- patient experience or recur^*^ or relapse or time or metastatic^*^ or progress^*^
- psycholog^*^ or psychosocial or experience^*^ or supportive care or social
- breast cancer or prostate cancer
- fear or anxiety or worry or shock.

### Inclusion Criteria

Articles were included if they: reported a study which explored the experience of any patients with a prostate or breast cancer recurrence (both local or distant recurrence were applicable, and data could have been collected at any time from directly after recurrence to end of life); used either quantitative or qualitative methodology to gather and analyse results; were published between January 1994 and April 2019; and were published in English.

### Exclusion Criteria

Articles were excluded if they: did not explicitly state that in their studies, participants had recurrent cancer and were subsequently included in data analysis. That is, studies may include participants with metastatic cancer which is not necessarily recurrent, hence these would be excluded. In addition, if no distinction is made between cancer types in analysis (i.e., breast or prostate cancer patients may be included in a study but analysed together with other cancers with no distinction) they were excluded.

### Screening Procedure

**Figure 1:**
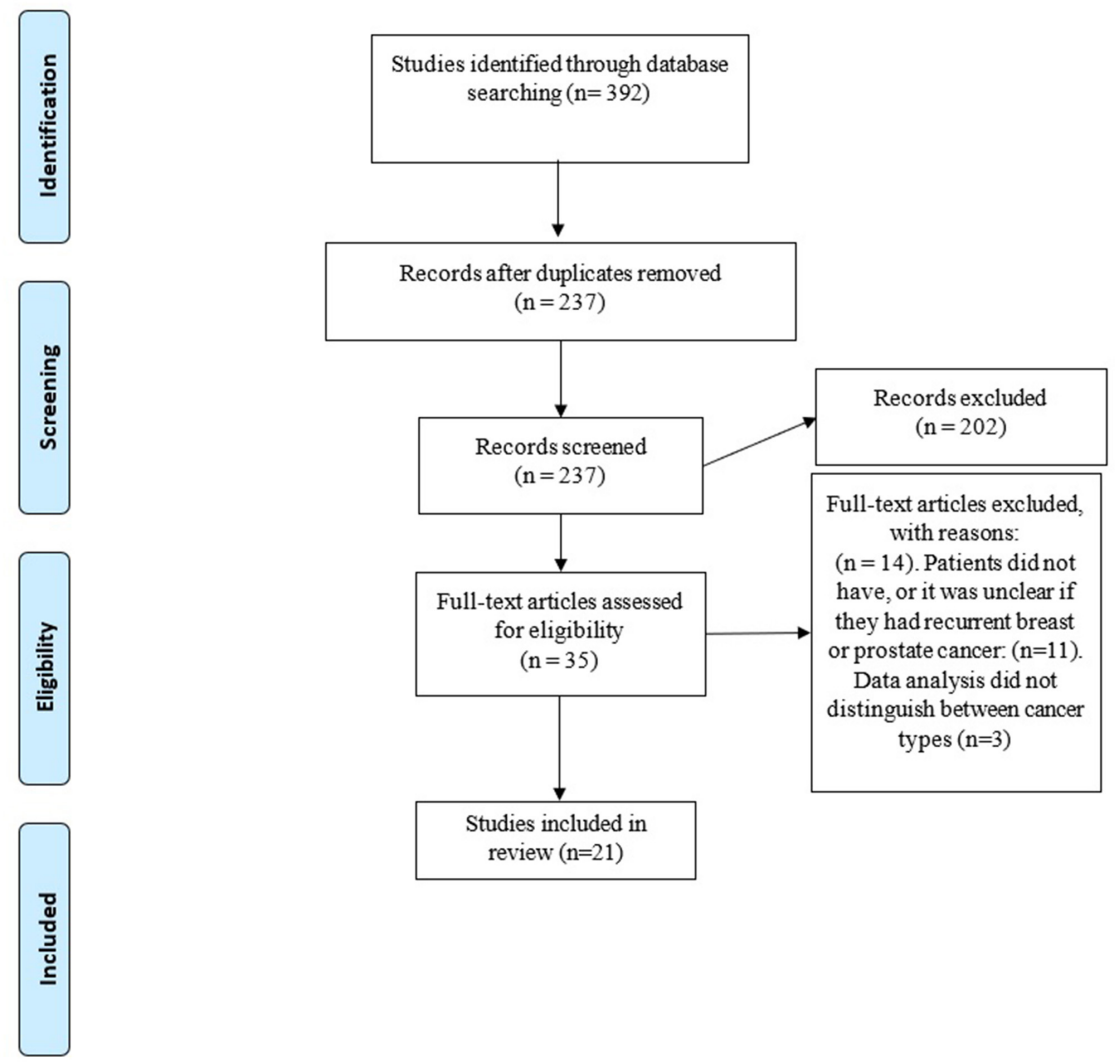
Flow diagram of database searching and study screening.

Two researchers (RJS, SC) independently screened articles that were identified through the database searches. First, titles and abstracts were screened, and non-relevant articles were excluded. Second, full articles of remaining studies were obtained and screened against this inclusion and exclusion criteria. Lastly, as a supplemental approach, reference lists of articles deemed to match the inclusion criteria were scanned. The procedure for database searching and study screening is outlined in Figure 1.

### Data Extraction

Extracted data included: sample characteristics; study aim and design; and cancer type and stage. Data were extracted by one researcher (RJS) and checked by a second (SC) for accuracy. Study quality and risk of bias were both independently assessed by two researchers (RJS, SC) using the Mixed Methods Appraisal Tool (MMAT) (Hong et al., 2018). The MMAT allows for the quality assessment of all study designs and is therefore suitable for this review. Any discrepancies in data extraction and quality appraisal were resolved through discussion.

### Data Abstraction and Synthesis

With consideration to the aim of the review as well as the heterogeneous character of eligible studies, there was limited scope for meta-analysis; instead, formal narrative synthesis was conducted with no minimum number of articles required. Using a convergent synthesis design (Hong et al., 2017), data from quantitative studies were combined with data from qualitative studies and were coded, and findings were categorised into themes based on the breakdown of different experiences. The outcomes synthesised in this review were measured either qualitatively or quantitatively by reliable and valid assessment tools and related to patient-reported levels of physical, psychological, and psychosocial indices of quality of life (QoL) that have impacted on the patients' experience of cancer recurrence. Themes related to the experience of prostate cancer patients with a cancer recurrence were compared to those of breast cancer patients with a cancer recurrence.

The precise timing of a recurrence will have a specific impact on the individual's health-related quality of life. This impact will differ between studies. If there is a comparison group alongside a recurrence group the difference between these will be used to judge the impact of recurrence. If there is no comparison group the impact of recurrence will be based upon scores from quantitative measures (if used by the authors). These measures will have scoring guidelines to judge what would be considered a normative or “standard” score. If there are qualitative findings with no comparison group these will be used to supplement results to build a wider comprehensive “picture” of the experience of patients at the time of recurrence. A within-subjects comparison can also be made where reference is made to previous assessments from patients at their primary diagnosis.

After the results have been presented from both cancer types, a comparison will take place. Any main similarities and differences will be outlined at this point and evaluating these will allow for judgement of if the subjective experience of recurrent breast cancer is broadly similar or different to that of prostate cancer, i.e., if several findings emerge in breast as well as prostate studies this would perhaps suggest a similar experience, whereas differing results would possibly suggest a different experience. Due to the outlined physical manifestations of breast and prostate cancer more credence will be given to the psychological and psychosocial concerns at this time when considering this comparison.

## Results

Overall, 392 articles were identified by the search strategy, of which 21 met inclusion criteria (Figure 1). Each article reported one relevant unique study.

### Description of Studies

Included articles were published between 1996 and 2017. Ten were conducted in the USA; three in Sweden; two in Japan; and one each in Australia, Finland, Israel, Italy, the Republic of Ireland, and the UK. Table 1 summarises details of the breast cancer studies and Table 2 the prostate cancer studies. Fifteen articles that met inclusion criteria examined the patient experience of breast cancer recurrence, whereas six articles examined the patient experience of prostate cancer recurrence. Reporting of age differed throughout studies. For the studies examining the experience of breast cancer recurrence 11 reported mean ages, and these had an aggregate mean of 56.7 years old. Two studies reported a median age- one of 50 (Brady and Helgeson, 2000) and the other of 57 (Cleeland et al., 2014). The last two studies reported age ranges: one simply 75 years and younger (Hall et al., 1996) and the other an age range of 55–81 years old (Sarenmalm et al., 2009). It is important to note that three of these articles used the same sample of participants, but for slightly different research aims- as such any findings highlighted in this review will be referenced to which particular article they came from (Sarenmalm et al., 2007, 2008, 2009). Of those studies examining the experience of prostate cancer recurrence, four (Pietrow et al., 2001; Ullrich et al., 2003; Lehto et al., 2015; Maguire et al., 2017) reported mean ages, with an aggregate mean of 66.2; and the other two (Ames et al., 2008, 2011) each reported a median age of 76, respectively.

**Table 1:** Breast cancer studies included in review.

**References**	**Aim**	**Sample characteristics**	**Design**	**Outcome measures**	**Quality score**
1. Andersen et al. (2005)	To analyse patients' reactions to a recurrence of cancer	30 females, mean age = 52 (SD = 11.6)	Controlled Prospective Study	IES; POMS; CES-D-SF; SF-36; SNI; PSS-Fa; PSS-Fr; DAS; KPS; SWOG rating scale.	^****^
2. Brady and Helgeson (2000)	To explore the relationship between social support and adjustment after a recurrence of breast cancer.	41 females, median age = 50	Quantitative	Adapted social support questions; BSI; COPE inventory	^****^
3. Bull et al. (1999)	Clarify relationship between recurrent breast cancer and quality of life	69 females, mean age = 53.3 (SD = 8.89)	Longitudinal study	Specifically designed scales.	^****^
4. Cleeland et al. (2014)	To characterise symptom burden, activities of daily living, health-related quality of life and work-related ability in order to inform clinical trials and treatments.	152 females, median age = 57	Observational cohort study	MDASI; WPAI; RSCL	^***^
5. Cohen (2002)	To explore emotional distress and coping strategies in patients with primary breast cancer vs. patients with recurrent breast cancer	41 females, mean age = 62.3 (SD = 7.7)	Observational cohort study	SCL-90; WCQ	^*****^
6. Hall et al. (1996)	To explore psychological morbidity in recurrent breast cancer patients.	61 females, age = 75 and younger.	Qualitative	Semi-structured interview	^*****^
7. Northouse et al. (2002)	To assess the quality of life of patients and their family members after recurrence	189 females, mean age = 54 (SD = 11.2)	Cross-sectional study	SF-36; FACT	^*****^
8. Oh et al. (2004)	To explore the quality of life of breast cancer survivors after a recurrence	54 females, mean age = 59.5	Observational cohort study	SF-36; CES-D; PANAS; IES-R; RDAS; MOS-SSS; PTGI; SBI-15R; Specifically developed Meaning and Vulnerability Scale	^*****^
9. Okamura et al. (2000)	To study the prevalence of psychological distress and risk factors of these following recurrence of breast cancer.	55 females, mean age = 52 (SD = 9)	Cross-sectional study	Structured clinical interview; POMS	^*****^
10. Okamura et al. (2005)	To examine the prevalence of, and factors linked!!break with psychiatric disorders, and the impact on quality of life after recurrence.	50 females, mean age = 53 (SD = 10)	Cross-sectional study	Structured clinical interview; MAC scale; EPQ-R; EORTC QLQ-C30; EORTC QLQ-BR23	^****^
11. Sarenmalm et al. (2007)	To examine predictors of health-related quality of life in postmenopausal women with recurrent breast cancer.	56 females, mean age = 65	Cross-sectional study	MSAS; HADS; SOC-13; EORTC QLQ-C30; IBCSG QoL	^****^
12. Sarenmalm et al. (2008)	To explore the symptom experience and predictors of distress and quality of life in women with recurrent breast cancer [the same sample as Sarenmalm et al., 2007 was assessed].	56 females, mean age = 65	Longitudinal study	MSAS; HADS; EORTC QLQ-C30	^****^
13. Sarenmalm et al. (2009)	To assess the main concerns of women with recurrent breast cancer, and how they were dealing with their situations (this sample was derived from the earlier Sarenmalm et al. studies).	20 females, age range 55–81	Qualitative	Semi-structured interview	^*****^
14. Thornton et al. (2005)	To clarify the effects of being diagnosed with cancer for a!!break second time on health-related quality of life.	140 females, mean age = 53 (SD = 9.7)	Prospective data extracted from larger Randomised Control Trial	SF-36	^***^
15. Turner et al. (2005)	To define the key emotional concerns of women newly diagnosed with recurrent or metastatic breast cancer.	68 females, mean age = 54.7 (SD = 13.5)	Mixed Methods	Semi-structured interview; HADS; IES; CARES-SF	^****^

*BSI, Brief Symptom Inventory; CARES-SF, Cancer Rehabilitation Evaluation System-short form; CES-D, Center for Epidemiological Studies-Depression; COPE, Coping Orientation to Problems Experienced; DAS, Dyadic Adjustment Scale; EORTC QLQ-C30 (BR23), European Organization for the Research and Treatment of Cancer Quality of Life Questionnaire (breast cancer specific); FACT, Functional Assessment of Cancer Therapy; HADS, Hospital Anxiety and Depression Scale; IBCSG- QoL, International Breast Cancer Study Group- Quality of Life; IES, Impact of Events Scale (R)(Revised); KPS, Karnofsky Performance Scale; MAC, Mental Adjustment to Cancer; MOS-SSS, Medical Outcomes Study-Social Support Scale; MSAS, Memorial Symptom Assessment Scale, PANAS, Positive and Negative Affect Schedule; POMS, Profile of Mood States; PSS-(fa; fr), Perceived Social Support (family; friends); PTGI, Posttraumatic Growth Inventory; RDAS, Revised Dyadic Adjustment Scale; RSCL, Rotterdam Symptom Checklist; SBI-15R, System of Belief Inventory; SCL-90, Symptom Checklist; SF-36, Short Form Health Survey; SOC-13, Sense of Coherence Scale; SNI, Social Network Index; SWOG, Southwest Oncology Group; WCQ, Ways of Coping Questionnaire; WPAI, Work Productivity and Activity Impairment*.

*** *refers to meeting 3 out of 5 quality criteria*,

**** *is 4 out of 5, and*

***** *is 5 out of 5*.

**Table 2:** Prostate cancer studies included in review.

**References**	**Aim**	**Sample**	**Design**	**Outcome measures**	**Quality rating**
1. Ames et al. (2008)	To appraise the psychological needs of men with a biochemical recurrence of prostate cancer	28 males, median age=76	Mixed Methods	Semi-structured focus group; FACT-P; SF-36; MAX-PC; POMS-B; LES; PSS	^***^
2. Ames et al. (2011)	To evaluate the acceptability effect size of a quality of life intervention for men with a biochemical recurrence of prostate cancer	57 males, median age = 76	Pilot study of randomised controlled trial	FACT-P; SF-36; MAX-PC; PSS-10; POMS-B	^***^
3. Lehto et al. (2015)	To investigate experiences and psychological well-being in prostate cancer patients who received various types of treatment.	74 males, mean age = 67	Cross-sectional study	Specifically designed survey; RSCL; SWLS; IIEF	^*****^
4. Maguire et al. (2017)	To examine the associations between prostate cancer survivors' treatment appraisals and fear of recurrence.	1,229 males (222 had recurrence), mean age = 68.48 (SD = 7.87)	Cross-sectional study	EORTC QLQ-C30; Fear of recurrence scale; DRS	^*****^
5. Pietrow et al. (2001)	To define the impact of PSA recurrence on health-related quality of life radical retropubic prostatectomy.	88 males, mean age = 63.4	Observational cohort study	SF-36; UCLA-PCI	^****^
6. Ullrich et al. (2003)	To compare cancer fear and mood disturbance after biochemical recurrence of prostate cancer with those without recurrence.	45 males, mean age = 66.1 (SD = 6.4)	Observational cohort study	AUA Symptom Index; Previously used Cancer Fear questions; POMS	^****^

*AUA, American Urological Association; DRC, Decisional Regret Scale; EORTC QLQ-C30, European Organization for the Research and Treatment of Cancer Quality of Life Questionnaire; FACT-P, Functional Assessment of Cancer Therapy- Prostate; IIEF, International Index of Erectile Function; MAX-PC, Memorial Anxiety Scale-Prostate Cancer; LES, Life Experiences Survey; POMS (B) Profile of Mood States (Brief); PSS-10 Perceived Stress Scale; RSCL, Rotterdam Symptom Checklist; SF-36, Short Form Health Survey; SWLS, Satisfaction With Life Scale; UCLA-PCI, University of California Los Angeles-Prostate Cancer Index*.

*** *refers to meeting 3 out of 5 quality criteria*,

**** *is 4 out of 5, and*

***** *is 5 out of 5*.

#### Study Methods

Of those studies examining the experience of breast cancer all but three were conducted with quantitative methods; with two using qualitative methods (Hall et al., 1996; Sarenmalm et al., 2009) and the other utilising mixed methods (Turner et al., 2005). One study (Ames et al., 2008) utilised mixed methods to examine the experience of prostate cancer, the remainder were conducted quantitatively. The research aims of included studies are described in Tables 1, 2.

#### Themes

Themes that emerged during data analysis were assigned to three broad categories: physical, psychological, and psychosocial issues. For ease of comparison between cancer types the main findings that emerged in included studies are outlined in Table 3, but more detail is described below.

**Table 3:** Common patient-reported issues after cancer recurrence.

**Breast cancer**			**Prostate cancer**		
**Physical**	**Psychological**	**Psychosocial**	**Physical**	**Psychological**	**Psychosocial**
Fatigue	Anxiety	Low QoL	Fatigue	Anxiety	Low QoL
Urination problems	Depression	Issues with medical staff	Urination problems	Depression	Issues with medical staff
Sexual problems	Stress	Importance of social support	Sexual problems	Frustration	Importance of social support
Shortness of breath	Emotional distress	Poor social functioning	Loss of muscle strength	Fluctuating mood	–
Poor appetite	Worrying	Unable to fulfil daily activities	Hot flushes	Anger	–
Taste change	Sadness	–	Incontinence		–
Weight loss	Irritability	–	–	–	–
Mouth sores	–	–	–	–	–
Dry mouth	–	–	–	–	–
Pain	–	–	–	–	–
Nausea and vomiting	–	–	–	–	–
Drowsiness	–	–	–	–	–
Limb Swelling	–	–	–	–	–
Numbness	–	–	–	–	–
Dizziness	–	–	–	–	–
Difficulty concentrating	–	–	–	–	–
Feeling bloated	–	–	–	–	–
Constipation	–	–	–	–	–
Diarrhoea	–	–	–	–	–
Coughing	–	–	–	–	–
Sweating	–	–	–	–	–

### Breast Cancer

#### Physical Issues

Physical symptoms experienced by breast cancer patients with a recurrence included: fatigue; sweats; coughing; a lack of appetite; dry mouth; pain; nausea and vomiting; drowsiness; swelling of limbs; numbness, feeling bloated; dizziness; taste change; problems with sex; constipation; diarrhoea; issues with urination; mouth sores; weight loss; shortness of breath; and difficulty concentrating (Turner et al., 2005; Sarenmalm et al., 2007, 2008; Cleeland et al., 2014). Furthermore, one study (Northouse et al., 2002) found that, in comparison to cancer patients in general, those with a recurrence rated their overall physical health lower. Further, patients' perceptions of their physical health at recurrence were found to be lower compared to: pre-recurrence (Bull et al., 1999); primary diagnosis (Andersen et al., 2005; Thornton et al., 2005); cancer patients in general (Northouse et al., 2002); and both population norms and disease-free breast cancer survivors (Oh et al., 2004). One study (Thornton et al., 2005) found that perceptions of physical health of women with distant recurrence were rated significantly lower than women with local recurrence.

#### Psychological Issues

Psychological problems were common among those with a breast cancer recurrence (Northouse et al., 2002; Turner et al., 2005). In a qualitative study (Hall et al., 1996), half of the sample (of a total of 38) were found to be clinically depressed or anxious (or both). Okamura and colleagues (Okamura et al., 2000) reported that 42% of their participants met criteria for major depressive disorder or adjustment disorders; with the prevalence rate of major depressive disorder akin to that found in patients after a primary diagnosis of cancer. However, a later study (Okamura et al., 2005) found the prevalence rate of psychiatric disorders to be lower, at 22% of their sample of recurrent breast cancer patients. Further, one study (Oh et al., 2004) found that in their sample, women with recurrent breast cancer did not suffer from clinical depression, prior to or following recurrence. There were different negative emotions experienced by those with a recurrence: high cancer-related stress (Andersen et al., 2005); emotional distress (Bull et al., 1999); general stress; worry; sadness; and irritability (Sarenmalm et al., 2007, 2008, 2009). Though another study (Oh et al., 2004) found that patients generally had good overall mood, as well as low levels of cancer-specific stress. A qualitative study (Sarenmalm et al., 2009) reported that participants often viewed recurrence as more distressing that their initial cancer diagnosis; but one study (Andersen et al., 2005) reported that patients' stress was equivalent at initial diagnosis as it was at recurrence, and another (Oh et al., 2004) reported some patients felt it was more stressful but others did not. Findings from one study (Cohen, 2002) suggested that, in comparison to women with primary breast cancer, women with local or metastatic recurrence displayed higher levels of depression, anxiety, and somatisation.

#### Psychosocial Issues

Self-reported overall QoL was negatively impacted by the diagnosis of a recurrence: in comparison to pre-recurrence (Bull et al., 1999; Andersen et al., 2005; Thornton et al., 2005); and compared to those with an early-stage primary diagnosis of cancer (Northouse et al., 2002). Issues with medical staff were reported; satisfaction with medical professionals was found to be fairly low (Bull et al., 1999; Turner et al., 2005). Furthermore, several patients in the study by Turner et al. (2005) expressed frustration at the method in which the diagnosis was given, and over 40% of their sample felt that there had been too long a delay between their reporting of concerning symptoms and the subsequent action by medical professionals leading to diagnosis of recurrence. Thirty out of 38 patients in one study (Hall et al., 1996) claimed to have received no support whatsoever from their hospital following recurrence.

Patients were concerned about their loss of independence and the impact on family members (Turner et al., 2005), and limitations to their social roles (Northouse et al., 2002; Thornton et al., 2005). Cleeland et al. (2014) reported several patients faced impairment with daily activities as well as issues with missing work and impairment when they were able to work. Social functioning (the ability to fulfil social roles) was found to be negatively impacted by recurrence (Bull et al., 1999; Northouse et al., 2002; Andersen et al., 2005; Thornton et al., 2005). Some patients described the good quality of their interpersonal relationships (Oh et al., 2004; Andersen et al., 2005). Brady and Helgeson (2000) examined the correlations between social support and adjusting to breast cancer recurrence. They found that emotional support from a partner and communicative support from an oncologist were correlated with fewer physical issues, but not to psychological distress. Further, psychological distress was related to decreased emotional support from a partner. Findings from the qualitative study by Sarenmalm et al. (2009) suggest that re-examining and altering social relationships was found to be a method of adjusting to cancer recurrence, and distress was lessened by receiving reassurance in regards to fears and uncertainty. Patients from this study found importance in changing their expectations from being cured, focussing on the quality of life rather than quantity and concentrating on the present rather than the past or future. An interesting finding from one study (Cohen, 2002) suggested that women with recurrent breast cancer were significantly less likely to use the adoption of a positive attitude as a coping mechanism than women with a primary diagnosis.

### Prostate Cancer

#### Physical Issues

For recurrent prostate cancer patients, problems with sexual activity were reported (Pietrow et al., 2001; Ames et al., 2008; Lehto et al., 2015), such as sexual dysfunction and low libido. Patients also had issues with experiencing hot flushes from their treatment, frequent urination and incontinence, fatigue, as well as loss of muscle strength (Ames et al., 2008; Maguire et al., 2017). Patients suffered pain, as well as reporting low levels of physical well-being (Ames et al., 2008, 2011).

#### Psychological Issues

Patients commonly reported high levels of anxiety (Ames et al., 2008; Lehto et al., 2015) due not only to the recurrence itself, but to PSA testing and subsequent results and related to their physical issues. Some patients reported anger and bitterness (Ames et al., 2008; Lehto et al., 2015) regarding their situation, as well as a frustration at the lack of a cure. One study (Lehto et al., 2015) described patients with depressive thoughts and fluctuating mood that were more pronounced than general prostate cancer patients. Though, Ames et al. (2011) found generally, participants had relatively low levels of anxiety, stress, and mental health issues, as well as reasonably raised mood. Moreover, an inconsistent picture emerged in the study by Ames et al. (2008) wherein participants rated their mood as high when measured qualitatively, which contrasted when measured quantitatively. Ullrich et al. (2003) found that recurrence in itself was not associated with greater mood disturbance or cancer-related fear. However, when patients with recurrence also had urinary symptoms they displayed high psychological distress; suggesting that these symptoms may be a more important factor.

#### Psychosocial Issues

Issues that arose in the study by Lehto et al. (2015) related broadly to the relationship between patients and their healthcare professionals. Several patients felt unhappy with the information given to them at diagnosis of recurrence. Some reported dissatisfaction at the way in which they learned of their condition in that some felt it too impersonal. Others deemed the behaviour and communication of healthcare professionals to be unsatisfactory, and half of their participants reported unhappiness with the care received (Lehto et al., 2015); however, experiences varied between the treatments undertaken. Maguire et al. (2017) noted that most of their sample were satisfied with the information they received about their condition and largely felt low regret over their choices regarding treatment. The participants in one (Ames et al., 2008) study reported generally good relationships with their doctors. In terms of social relationships, participants in the same study reported the maintenance of good social relationships as an important marker of their QoL, and social support from friends and family was commonly reported as a useful method of coping with the cancer (Ames et al., 2008; Lehto et al., 2015). In the study by Lehto et al. (2015) most participants regarded their condition as having no effect on the relationship with their partner. One study (Ames et al., 2008) found that men with a recurrence of prostate cancer had worse health-related and prostate cancer-specific QoL than patients without recurrence, though the general QoL of recurrent patients in this study was higher than patients with other chronic illnesses. Pietrow et al. (2001) found small negative differences in health-related QoL in patients with recurrence vs. those without, but deemed overall QoL to be very similar in these two groups.

### Comparison Between Breast and Prostate Cancer

Despite differences in the physical manifestation of breast and prostate cancer, some physical symptoms were highly prevalent in both types of cancer: pain; fatigue; problems with sexual activity; and bowel and bladder issues. Psychological morbidity was common for both cancer types. Some negative emotions, common with either type of cancer recurrence, were: sadness, worry, irritability, anxiety, uncertainty, and stress. Several, though not the majority of patients of both cancer types expressed dissatisfaction with medical professionals. The importance of social relationships as a means of emotional support was commonly reported across both cancer types. Noting differences is complex due to the disparity in the number of breast and prostate cancer studies. For example, as opposed to breast cancer (Okamura et al., 2000, 2005), no studies assessed prostate cancer patients for formal criteria of psychological disorders. More physical problems were associated with breast cancer recurrence, though the above issue may in part account for this.

### Quality Appraisal

The MMAT includes five criteria of quality to judge studies (Hong et al., 2018). Included studies' quality scores ranged from meeting three out of the five criteria to meeting all five criteria. These criteria differ based upon the design of each study. Most studies were found to be of moderate quality. Of the 15 studies with a quantitative design it was observed that quality differed, with only five judged to meet all five criteria (Okamura et al., 2000; Northouse et al., 2002; Oh et al., 2004; Lehto et al., 2015; Maguire et al., 2017). The two studies with a qualitative design (Hall et al., 1996; Sarenmalm et al., 2009) were judged to meet all five criteria. The two studies with mixed-methods methodology were judged to only meet three criteria (Turner et al., 2005; Ames et al., 2008). An issue with both of these studies was that the authors did not outline explicitly how each research component integrated with the other. Many studies had small sample sizes as well as being at risk of non-response bias, which lowered the generalisability of the results. Table 4 contains full details of the quality assessment of the included studies; and for ease of comparison, quality scores are displayed in Tables 1, 2 alongside study details.

**Table 4:** Quality appraisal.

**Qualitative**	**Is the qualitative approach appropriate to answer the research question?**	**Are the qualitative data collection methods adequate to address the research question?**	**Are the findings adequately derived from the data?**	**Is the interpretation of results sufficiently substantiated by data?**	**Is there coherence between qualitative data sources, collection, analysis and interpretation?**
Hall et al. (1996)	Yes	Yes	Yes	Yes	Yes
Sarenmalm et al. (2009)	Yes	Yes	Yes	Yes	Yes
**Quantitative randomised controlled trials**	**Is randomisation appropriately performed?**	**Are the groups comparable at baseline?**	**Are there complete outcome data?**	**Are outcome assessors blinded to the intervention provided?**	**Did the participants adhere to the assigned intervention?**
Ames et al. (2011)	Can't tell	Yes	Yes	No	Yes
**Quantitative non-randomised**	**Are the participants representative of the target population?**	**Are measurements appropriate regarding both the outcome and intervention (or exposure)?**	**Are there complete outcome data?**	**Are the confounders accounted for in the design and analysis?**	**During the study period, is the intervention administered (or exposure occurred) as intended?**
Andersen et al. (2005)	Can't tell	Yes	Yes	Yes	Yes
Cleeland et al. (2014)	Yes	Yes	No	Yes	No
Cohen (2002)	Yes	Yes	Yes	Yes	Yes
Northouse et al. (2002)	Yes	Yes	Yes	Yes	Yes
Oh et al. (2004)	Yes	Yes	Yes	Yes	Yes
Pietrow et al. (2001)	Yes	No	Yes	Yes	Yes
Ullrich et al. (2003)	Yes	No	Yes	Yes	Yes
**Quantitative descriptive**	**Is the sampling strategy relevant to address the research question?**	**Is the sample representative of the target population?**	**Are the measurements appropriate?**	**Is the risk of non-response bias low?**	**Is the statistical analysis appropriate to answer the research question?**
Brady and Helgeson (2000)	Yes	Yes	No	Yes	Yes
Bull et al. (1999)	Yes	Yes	Yes	No	Yes
Lehto et al. (2015)	Yes	Yes	Yes	Yes	Yes
Maguire et al. (2017)	Yes	Yes	Yes	Yes	Yes
Okamura et al. (2000)	Yes	Yes	Yes	Yes	Yes
Okamura et al. (2005)	Yes	Yes	Yes	No	Yes
Sarenmalm et al. (2007)	Yes	Yes	Yes	No	Yes
Sarenmalm et al. (2008)	Yes	Yes	Yes	No	Yes
Thornton et al. (2005)	Yes	Yes	Yes	No	No
**Mixed methods**	**Is there an adequate rationale for using a mixed methods design to address the research question?**	**Are the different components of the study effectively integrated to answer the research question?**	**Are the outputs of the integration of qualitative and quantitative components adequately interpreted?**	**Are divergences and inconsistencies between quantitative and qualitative results adequately addressed?**	**Do the different components of the study adhere to the quality criteria of each tradition of the methods involved?**
Ames et al. (2008)	No	No	Yes	Yes	Yes
Turner et al. (2005)	Yes	No	Yes	No	Yes

## Discussion

From the available evidence, there appears to be several similarities in the experience of recurrent breast and prostate cancer. Moreover, most disparities appear within cancer types, with mixed results for certain outcomes across studies. The reported psychological factors indicate the biggest differences between studies (and not between cancer types). It is worth consideration that this could be in part related to the different outcome measures used to capture the experience of recurrent cancer. This is perhaps best demonstrated by the disparity already identified within Ames et al. (2011), wherein participants rated their mood highly when measured qualitatively but low when measured quantitatively.

The prostate cancer study (Ames et al., 2011) that reported generally positive mood of patients with recurrence was rated moderately, meeting 3 out of 5 quality criteria. The same rating was given to the study (Ames et al., 2008) where participants' mood rated high when measured qualitatively, but not quantitatively. Little difference in QoL between patients with recurrence and those with primary diagnosis was found by Pietrow et al. (2001), and this study was judged to meet 4 out of 5 quality criteria; a rating also given to the study (Ullrich et al., 2003) which found that recurrence in itself was not a significant factor on cancer fear and mood disturbance. However, fear was considered higher in patients with recurrence than without in the study by Maguire et al. (2017). This set of results initially suggests that the quality of studies may be important in interpreting results. However, the findings from the breast cancer studies may counter this opinion with one study (Oh et al., 2004) finding generally good mood and low levels of cancer specific-stress. This particular study was judged to meet all 5 quality criteria.

As this review was not examining the efficacy of a treatment or intervention, but rather examining the experiences of included patients, the process of distinguishing between RCTs and other study designs, in terms of levels of evidence, would not be as pertinent as it may otherwise be. Hence, the study featuring an RCT was a prostate cancer study (Ames et al., 2011) and diverged most from the other prostate cancer studies. It is interesting that this was a pilot study and therefore had a relatively small sample size. Inconsistent results were found within other study designs which suggests therefore that these design features do not necessarily explain differences found between studies.

The articles reviewed infer that gender may not explain differences in the recurrence experience. Interestingly, a recent meta-analysis suggests that fear of cancer recurrence is stronger in women than men, but whether this applies to emotional distress after actually experiencing a recurrence is unclear from this review. There is some suggestion that gender plays a role in how primary cancer is experienced (Pud, 2011; Linden et al., 2012); however, the literature is mixed in that some research has found little difference or inconsistent results in relation to various aspects of the cancer experience between genders (Miaskowski, 2004; Garrett et al., 2011; Ahmed et al., 2017).

It has been suggested that the fear of cancer recurring decreases with age (Lim and Humphris, 2020), so that younger cancer survivors will be more concerned about this possibility. This is plausibly explained by younger people having a longer life expectancy. In the current review breast cancer patients with recurrence were generally younger than those with prostate cancer, and so this could apply to the lived experience of recurrence rather than just the fear. However, with minimal difference found between the cancer types this suggestion is not supported.

In summary, findings from this review point to differences in the recurrent cancer experience being based upon individual factors, rather than having either recurrent breast or recurrent prostate cancer. There is evidence in this review to support this interpretation. As indicated previously, social support was important to patients at the time of recurrence. Previous research (Yoo et al., 2017) has found a link between higher perceived social support and higher quality of life and lower depressive symptoms among patients with a primary diagnosis of cancer, thus logically this may apply at the time of recurrence. Further, there was indication that treatment received may be an important factor in quality of life. It has been suggested that differing treatment in primary prostate cancer patients led to different physical problems (Bacon et al., 2001), and this could therefore subsequently impact on psychological well-being. Thus, it is possible that the experiences of patients may differ based on factors such as these, and would be would be worthy of further investigation.

### Comparison to Previous Research

Within the literature, it is firmly established that fear of cancer recurrence, as well as an actual recurrence of cancer, are sources of emotional distress (Simard et al., 2013; Schouten et al., 2019); as such, this review is consistent with findings from the meta-ethnography carried out by Wanat et al. (2016) and the earlier narrative review by Vivar et al. (2009), which both described a wide range of negative issues that accompany cancer recurrence. This review adds to this research by conducting a comparison of cancer types, based upon the available literature. As noted, breast and prostate cancer were chosen as they differ in a number of ways, not least as they effect males and females (almost) exclusively, but future research could be designed to capture a wider range of cancer types than just breast and prostate cancers. Such research would help to clarify these findings.

### Limitations

Though the review was exploratory in nature, the cancer type comparison conducted should be read with the caveat that there were far fewer studies included examining the experience of patients with recurrent prostate cancer as opposed to breast cancer. All prostate cancer studies were quantitative, and whilst the integrative nature of this review means the study design is less important, it is perhaps indicative of the relative lack of research into the experience of recurrent prostate cancer patients. As such, there were some aspects of the patient experience that were measured solely in breast cancer patients and therefore cannot be compared. Whilst a gender difference is an interesting comparison point, with the two cancer types selected it is not possible to delineate between cancer type and gender as factors in how cancer recurrence is experienced, this is a major limitation of the review. This is partially offset by being only one of a number of factors discussed, but to further distinguish between gender and cancer type it would be beneficial in any future comparison to include another cancer type that affects men and women on a fairly equal proportion. In addition, several of the studies were not primarily exploring the experience of patients with recurrent cancer but had some patients who had recurred included in their analysis. Another limitation is the variety of timing when patients were investigated. For example, there were different time points when data were collected, as well as the time between initial diagnosis and recurrence varying across studies.

### Recommendations

An exploratory, longitudinal study directly comparing cancer types at the time of a recurrence would greatly add to the findings of this review. Ensuring high methodological quality of such research would address concerns raised in this review. This review has touched on factors that may result in lower quality of life in recurrent cancer patients (such as age, disease stage, and treatment received) that were not easily compared here. As such these could be explored as moderating variables in this new suggested research.

### Clinical Implications

Healthcare professionals may find this review of assistance to clarify what patients may experience at the time of a cancer recurrence with two prevalent cancer types. It was demonstrated that between these cancers, the experience of cancer recurrence might have many similarities, and as such due consideration is needed toward the care and support of the individual at the time of a cancer recurrence.

## Conclusions

This review primarily sought to identify if, based on evidence from the published literature, the type of cancer a patient had at the time of a recurrence had an impact on how cancer recurrence is experienced- based upon physical, psychological, and psychosocial indices of QoL in recurrent breast and prostate cancer patients. It highlights the multifarious issues created for cancer patients at the time of a cancer recurrence, thereby building upon findings from such previous research (Vivar et al., 2009; Wanat et al., 2016). Based upon the comparison conducted, findings suggest that it is likely that any differences in the experience of recurrent cancer are more heavily influenced by individual factors, rather than cancer type, though concerns have been raised about available study quality and differing outcome measures in this interpretation. Adding to the literature, this review is the first to specifically explore and compare the experience of patients with recurrent prostate or breast cancer; the most common cancers in males and females, respectively. As such, it has been possible to explore potential reasons for differences in experience.

## Data Availability Statement

The original contributions presented in the study are included in the article/supplementary material, further inquiries can be directed to the corresponding author/s.

## Author Contributions

RS, SC, JD, and GH conceived of the review. RS and SC were responsible for study screening and quality assessment. RS conducted data analysis and drafting of the manuscript. SC, JD, and GH made important revisions to the paper. All authors contributed to the article and approved the submitted version.

## Conflict of Interest

The authors declare that the research was conducted in the absence of any commercial or financial relationships that could be construed as a potential conflict of interest.
